# Conventional Cytogenetic Analysis and Array CGH + SNP Identify Essential Thrombocythemia and Prefibrotic Primary Myelofibrosis Patients Who Are at Risk for Disease Progression

**DOI:** 10.3390/ijms25074061

**Published:** 2024-04-05

**Authors:** Joseph Tripodi, Ronald Hoffman, Douglas Tremblay, Daiva Ahire, John Mascarenhas, Marina Kremyanskaya, Vesna Najfeld

**Affiliations:** 1 Tumor CytoGenomics Laboratory, Department of Pathology, Molecular and Cell-Based Medicine, Icahn School of Medicine at Mount Sinai, New York, NY 10029, USA; 2 Tisch Cancer Institute, Icahn School of Medicine at Mount Sinai, New York, NY 10029, USA

**Keywords:** Philadelphia chromosome-negative myeloproliferative neoplasms, essential thrombocythemia, prefibrotic primary myelofibrosis, conventional cytogenetics, Comparative Genomic Hybridization + Single Nucleotide Polymorphism array, MPN disease progression

## Abstract

The Philadelphia chromosome-negative myeloproliferative neoplasms (Ph-MPNs) are a heterogeneous group of clonal hematopoietic malignancies that include polycythemia vera (PV), essential thrombocythemia (ET), and the prefibrotic form of primary myelofibrosis (prePMF). In this study, we retrospectively reviewed the karyotypes from conventional cytogenetics (CC) and array Comparative Genomic Hybridization + Single Nucleotide Polymorphism (aCGH + SNP) in patients with ET or prePMF to determine whether the combined analysis of both methodologies can identify patients who may be at a higher risk of disease progression. We performed a comprehensive genomic review on 169 patients with a clinical diagnosis of ET (154 patients) or prePMF (15 patients). Genomic alterations detected by CC or array-CGH + SNP were detected in 36% of patients. In patients who progressed, 68% had an abnormal genomic finding by either technology. There was a shorter progression-free survival (PFS) among patients who were cytogenetically abnormal or who were cytogenetically normal but had an abnormal aCGH + SNP result. Leveraging the ability to detect submicroscopic copy number alterations and regions of copy neutral-loss of heterozygosity, we identified a higher number of patients harboring genomic abnormalities than previously reported. These results underscore the importance of genomic analysis in prognostication and provide valuable information for clinical management and treatment decisions.

## 1. Introduction

The Philadelphia chromosome-negative myeloproliferative neoplasms (Ph-MPNs) are a heterogeneous group of clonal hematopoietic neoplasms that include polycythemia vera (PV), essential thrombocythemia (ET) and the prefibrotic form of primary myelofibrosis (prePMF) [1]. ET and prePMF are each characterized by thrombocytosis and are difficult to distinguish by clinical and histopathological criteria. ET and prePMF also share overlapping cytogenetic abnormalities with other Ph-MPNs, including a gain of chromosome 1q, trisomy of chromosomes 8 and 9, as well as del(13q) and del(20q) [2,3]. While chromosomal abnormalities are detected in 50% of patients with PMF and in ~30% with PV, they are relatively rare in the context of ET with an incidence of <10%. Approximately 50–60% of ET patients harbor driver mutations in *JAK2*, 20–25% in *CALR*, and 2–3% in *MPL*, while 10–16% are triple negative. Ph-MPNs have a variable risk of progressing to more overt forms of the disease and to accelerated or blast phase MPN (MPN-AP/MPN-BP). MPN-AP is defined by the presence of 10% to 19% of myeloid blasts in the peripheral blood or bone marrow and greater than or equal to 20% in MPN-BP. Although the majority of patients with Ph-MPN do not experience disease progression, the cumulative lifetime risk is variable for patients with Ph-MPN, ranging from 1 to 4% for ET patients and 5–10% for prePMF to 14% for overt PMF patients [4,5,6,7]. 

The cause of disease progression has yet to be fully understood, making prognostication difficult. Many studies have identified risk factors for leukemic transformation such as advanced age, severe anemia, leukocytosis, circulating blasts >2%, thrombocytopenia, advanced bone marrow fibrosis, cytogenetic abnormalities and acquisition of ≥2 high-risk mutations [5,8,9,10,11]. Currently, prognostic scoring systems in ET incorporate gene mutations (IPSET-survival and MIPSS-ET) but not karyotype, which is in contrast to PMF, which relies on an abnormal karyotype to aid in prognostication (DIPPS+ and MIPSS70+ v.2) [12]. A recent study showed that an abnormal karyotype has prognostic relevance in patients with ET, although greater than 90% of patients will exhibit a normal karyotype at diagnosis [2]. Therefore, we retrospectively reviewed the karyotypes from conventional cytogenetics (CC) and array Comparative Genomic Hybridization + Single Nucleotide Polymorphism (aCGH + SNP) in patients with ET or prePMF to determine whether aCGH + SNP can increase the sensitivity of detecting genomic abnormalities and if the combined analysis of both testing methodologies can identify a group of patients who may be at risk of disease progression. Since ET and prePMF can each be characterized by thrombocytosis and can be distinguished only by subtle changes in marrow megakaryocytic morphology, we lumped together patients with these two entities in order to provide prognostic information to the clinical community that might be the most useful.

## 2. Results

### 2.1. Baseline Characteristics

As shown in Table 1, based on the WHO 2016 criteria, we identified 169 patients with a clinical diagnosis of ET (154 patients) or prePMF (15 patients); 96 were female (57%), and 73 were male (43%) with a median age of 62 years (range 13–92). Driver mutational analysis was performed in 166 patients, and *JAK2* mutations were present in 61% of cases, *CALR* in 21%, and *MPL* in 8% of cases. The remaining 10% of patients were triple negative. At the time of the initial study, an abnormal karyotype was observed in 16% of patients, and the remaining 84% were either normal (77%) or had non-diagnostic results (7%) due to failed or limited peripheral blood chromosome analysis. Of the 121 patients that were analyzed using aCGH + SNP, 8% had prior abnormalities as detected by conventional cytogenetic analyses, while abnormalities were found in an additional 31% (34/111). Among the 169 patients, 98 (58%) were treated with hydroxyurea, 26 (15%) with anagrelide, 17 (10%) with ruxolitinib, and 14 (8%) with various formulations of interferon, either as a single agent or as part of multiline line therapy, and there were 57 patients who were treatment naïve. 

### 2.2. Disease Progression

In our cohort, 30% of patients had disease progression. Progression to PV occurred in 6 patients (4%), 11 (7%) progressed to AP/BP, and 33 (19%) progressed to either MF, chronic myeloid leukemia or myelodysplastic syndrome. ET patients who progressed to PV did not fulfill the WHO 2016 criteria for PV since red cell mass was not performed, and these patients may represent masked PV. Disease progression, irrespective of its form, was associated with genomic alterations detected by aCGH + SNP, cytogenetic analysis, or both at the time of first encounter. In these 50 patients who progressed, 68% had an abnormal genomic finding by either technology compared to 32% who were normal (*p* < 0.001). Of patients who had disease progression, *JAK2*, *CALR*, and *MPL* mutations were observed in 62%, 24%, and 8%, respectively, with the remaining 6% being triple negative. Furthermore, 22% harbored additional adverse myeloid gene mutations (*SF3B1*, *SRSF2*, *U2AF1*, *TP53*) as identified by the mutation-enhanced international prognostic system for ET (MIPSS-ET). Out of the 57 patients who did not receive therapy, 19% (11/57) had disease progression to MF, PV, or MDS compared to 35% (39/112) who had at least one line of therapy. Among the 39 patients who received therapy and had disease progression, the majority had post-MPN MF (25 patients), and 11 patients progressed to AP/BP. 

### 2.3. Conventional Cytogenetic Results

**Figure 1:**
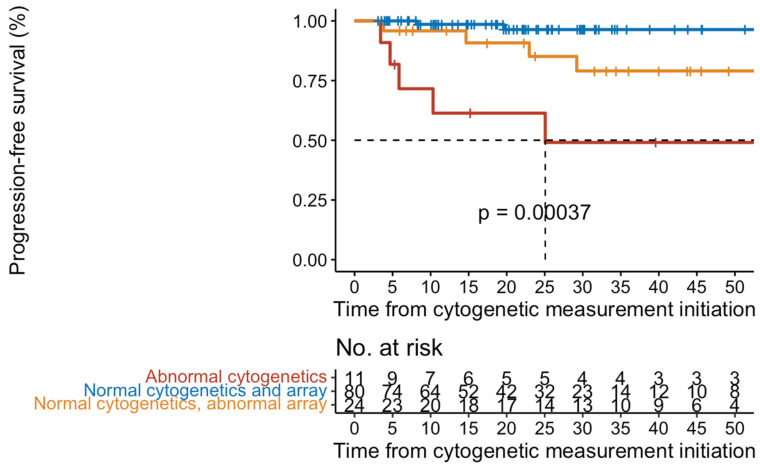
Kaplan-Meier curves of progression-free survival in 169 patients with ET/prePMF.

As shown in Table 2, a complex karyotype was observed in 3% of patients or 19% (5/27) of those with an abnormal karyotype. The most frequent chromosomal abnormalities observed were interstitial deletion of 13q (26%), interstitial deletion of chromosome 20q (15%), abnormalities of chromosome 12p (15%), and +9/9p, +8, and +1q observed in 3 patients each. Among those patients who had a cytogenetic abnormality, 63% progressed to either AP/BP or MF, compared to only 19% (24/125) who were cytogenetically normal (*p* < 0.001). There was no statistical difference in the frequency of cytogenetic abnormalities among patients who received therapy compared to those who were treatment naïve. However, as shown in Figure 1, there was a shorter progression-free survival (PFS) among patients who were cytogenetically abnormal with a median PFS of 25 months (95% CI 5.8-not reached) compared to cytogenetically normal patients (*p* < 0.001) (median PFS not reached) with a median follow-up of 32.7 months. 

### 2.4. Array-CGH + SNP Results

**Figure 2:**
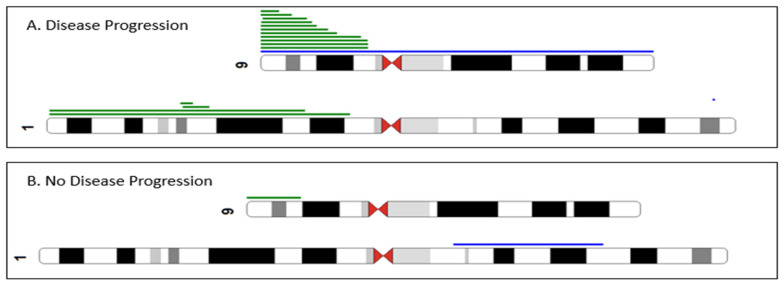
Partial ideogram summarizing the chromosomal abnormalities detected by array-CGH + SNP analysis. Green lines indicate regions of CN-LOH, and blue lines indicate gain. 9p and 1p CN-LOH were more frequent in ET/prePMF patients with disease progression (panel (**A**)) compared to patients without disease progression (panel (**B**)). We also performed a multivariable Cox regression analysis, and an abnormal array retained its significance associated with PFS (*p* = 0.018, HR = 2.89, 95% CI: 1.2, 6.97). In addition, abnormal cytogenetics retained its association with worsening progression-free survival (*p* = 0.010, HR = 2.35 95% CI: 1.23–4.51). There was no association with age, gender, or HMR mutations.

To identify cryptic genomic abnormities, aCGH + SNP was performed on 121 patients, of which 110 patients had normal karyotype or non-diagnostic results. Genomic abnormalities were observed in 36% of patients, and 64% were normal. In those 44 patients who were abnormal by aCGH + SNP, conventional cytogenetic abnormalities were observed in 10 patients (29%), while the remaining 34 patients were normal or had non-diagnostic cytogenetic results and only harbored aCGH + SNP abnormalities. As shown in Figure 1, there was a shorter progression-free survival among patients who were abnormal by aCGH + SNP but were cytogenetically normal (*p* < 0.001). In those patients that progressed, the most frequent abnormality was 9p copy-neutral loss of heterozygosity (CN-LOH), occurring in 11 patients (Figure 2).

## 3. Discussion

Genomic studies in the Ph-MPN, such as karyotype, driver gene mutational status, and the presence or absence of adverse or high molecular risk mutations, play a crucial role in disease prognostication. While an abnormal karyotype is infrequent in ET, occurring in less than 10% of cases, its prognostic relevance remains uncertain [13]. Although in a recent study, an abnormal karyotype in patients with ET was shown to be associated with shorter overall survival [2], we report here that an abnormal karyotype is associated with shorter progression-free survival compared to patients who are karyotypically normal.

In contrast to previous studies indicating cytogenetic abnormalities occur in less than 10% of cases, our study revealed cytogenetic abnormalities in 16% of patients. Furthermore, we demonstrate that the integration of conventional cytogenetics and aCGH + SNP increased the sensitivity of detecting genomic abnormalities from 16% (cytogenetics alone) to 36% when incorporating aCGH + SNP, underscoring the efficacy of a combined approach.

Array-CGH + SNP testing can detect cryptic sub-microscopic copy number alterations as well as regions of CN-LOH. CN-LOH results in duplication of a maternal or paternal chromosome or chromosomal region and concurrent loss of the other allele. In myeloid malignancies, CN-LOH has been associated with the duplication of oncogenic mutations with concomitant loss of the normal allele. Studies have shown that 9p CN-LOH, resulting in higher *JAK2*V617F allele burdens, has been reported in 6–18% of patients with ET and up to 57% in patients transformed from chronic ET, PV, or PMF to blast phase [14,15]. In this study, 9p CN-LOH was more frequent in patients who had disease progression (Figure 2). We also found that 1p CN-LOH occurred solely in patients with disease progression, resulting in higher *MPL* mutation allele burdens. Although we did not detect 19p CN-LOH in any of our 35 patients with *CALR* mutations, in a recent study, *CALR*-high patients, defined as a variant allele frequency (VAF) ≥60%, were found to be associated with shorter overall survival compared to CALR-low patients (VAF < 60%) [16].

Sequential genomic studies of MPN patients, including ET, have shown that the acquisition of genomic abnormalities may be associated with disease progression [17,18]. In this study, adverse mutations, according to the mutation-enhanced international prognostic systems for essential thrombocythemia (MIPSS-ET), were more frequent in patients who progressed (22%, 11/50) compared to those who did not have disease progression (3%, 3/115).

The combination of both conventional cytogenetics and aCGH + SNP increased the sensitivity of detecting genomic abnormalities from 16% (cytogenetics alone) to 36% when incorporating aCGH + SNP. These data indicate that genomic alterations detected by conventional cytogenetic analyses or CGH + SNP array can identify a subset of ET/prePMF patients that are at a higher risk of disease progression. Genomic alterations identified by aCGH + SNP and conventional cytogenetics are more frequent in patients with ET/prePMF who had disease progression, underscoring the importance of genomic analysis in prognostication and providing valuable information for clinical management and treatment decisions.

## 4. Materials and Methods

### 4.1. Patients

This study included a de-identified cohort of 169 patients diagnosed with ET or prePMF who were evaluated at our institution between November 2017 and August 2023. Study data were collected and managed using REDCap electronic data capture tools hosted at Icahn School of Medicine at Mount Sinai. Disease progression was defined as those patients who had fibrotic progression (MF) from the chronic forms of MPN, progression to other forms of MPN, or accelerated phase/blast phase (AP/BP), which is characterized by the presence of 10–19% or ≥20% of peripheral blood or bone marrow blasts, respectively.

### 4.2. Conventional Cytogenetics/Chromosomal Microarray

G-banded metaphase cells were obtained from bone marrow and/or unstimulated peripheral blood as per standard procedures. A high-resolution array comparative genomic hybridization (aCGH) platform using Agilent’s 2x400k CGH + SNP GenetiSure Cancer array (Agilent Technologies, Santa Clara, CA, USA) was performed on DNA extracted from bone marrow and/or unstimulated peripheral blood as previously described [19]. Array analysis was performed using Agilent’s CytoGenomics software version 5.2.1.4 (Agilent Technologies, Santa Clara, CA, USA). CNAs were filtered to exclude those <100 kb, nested aberrations, Y chromosome calls in females, and reference DNA CNVs. Regions of CN-LOH were called if they contained a minimum of 10 probes and were >10 Mb in size.

### 4.3. Next Generation Sequencing

Sequencing was carried out using a commercial CLIA-certified myeloid malignancy NGS panel (NeoGenomics, Inc., Fort Myers, FL, USA). The NeoGenomics NGS Myeloid Panel is a targeted next-generation sequencing (NGS) assay designed to detect genetic alterations such as single nucleotide variants (SNVs), copy number variants (CNVs), insertions and deletions (InDels) associated with myeloid neoplasms. This panel utilizes high-throughput sequencing technology to detect mutations and other genomic alterations in genes relevant to myeloid malignancies, including but not limited to myelodysplastic syndromes (MDS), myeloproliferative neoplasms (MPN), and acute myeloid leukemia (AML).

### 4.4. Statistics

Hypothesis testing was two-sided and conducted at the 5% level of significance. Statistical analyses were performed in RStudio software version 1.3.1056 (RStudio: Integrated Development for R. RStudio, PBC, Boston, MA, USA) using premade analytical packages, including tidyverse and survival.

## Figures and Tables

**Table 1:** Genomic characterization of 169 ET/prePMF patients.

Variables	All Patients (*n* = 169)	No Progression (*n* = 119)	Progression (*n* = 50)
Age in years, median (range)	62 (13–92)	56 (13–92)	67 (31–89)
Gender (female), *n* (%)	96 (57)	70 (59)	26 (52)
Cytogenomic Results			
Normal, *n* (%)	108 (64)	92 (77)	16 (9)
Abnormal, *n* (%)	61 (36)	27 (23)	34 (68)
Driver Mutations			
*N*, evaluable	n = 166	n = 116	n = 50
JAK2, *n* (%)	99 (60)	68 (59)	31 (62)
MPL, *n* (%)	13 (8)	9 (8)	4 (8)
CALR, *n* (%)	35 (21)	23 (20)	12 (24)
JAK2/CALR, *n* (%)	2 (1)	2 (2)	0 (0)
Triple Negative, *n* (%)	17 (10)	14 (12)	3 (6)
Next Generation Sequencing			
Adverse Molecular Risk (MIPSS-ET)			
*N*, evaluable	n = 165	n = 115	n = 50
1 gene, *n* (%)	13 (8)	3 (3)	10 (20)
≥2 gene, *n* (%)	1 (<1)	0 (0)	1 (2)

**Table 2:** Summary of 27 patients who were cytogenetically abnormal.

ID#	Age	Diagnosis	Gender	Tissue	Cytogenetics Karyotype	Array	Mutations	Progression to:
1	75	ET	M	BM	45,XY,add(4)(p14),der(5;17)(p10;q10),der(8)add(8)(p11.2)add(8)(q13),add(13)(p11.2),der(15)t(15;16)(p11.2;q11.2),-16,-18,add(19)(q13.3),del(21)(q21q22),+2mar[cp19]/ 46,XY[1].ish der(4)del(4)(p14p16)ins(4;8)(p14;q24.3q23)(MYC+),der(5;17)(p10;q10)(D17Z1+),der(8)(?::8p11.2->8q12::8q24.1->8q22::8q23->8q22::8q23->8q24.1::?)(D8Z2+,MYC++),der(13)(8qter::8q22->8q24.3::13p11.2->13qter)(MYC+)	ND	JAK2	AP/BP
2	55	ET	M	BM	47,XY,+Y,t(11;19)(q23;p13.1)[16]/ 46,XY[4]	ND	Triple Negative	AP/BP
3	74	ET	M	BM	42~43,X,-Y,add(5)(q11.2),add(7)(p13),?t(9;17)(q21;p13),-15,-16,der(19)t(15;19)(q11.2;p13.3),-21[cp3]/46,XY[17]	ND	JAK2	AP/BP
4	73	ET	F	PB	46,XX,del(12)(p12p13)[14]/47,idem,+8[2]/46,XX,t(1;9)(p13;q32),del(12)(p12p13)[cp2]/46,XX[3] ADDENDUM on 8/21/2019: 46,XX,del(12)(p12p13).ish t(7;12)(q36;p13)(5′ETV6+;3′ETV6+)	4q24(106036993_106456054)x1, 9p24.3p13.1(0_38694064)x2 hmz	JAK2	AP/BP
5	65	ET	F	BM	46,XX,der(5;19)(p10;q10),+19[5]/45,XX,der(5)t(5;9)(q13;p22),der(7)add(7)(p12)add(7)(q22),add(9)(p22),t(12;19)(p13;q13.1),-13,-15,add(17)(q21.3),+mar[4]/44,sl,-9,der(14)t(9;14)(q22;p24),der(16)t(9;16)(p22;p13.1)[2]/46,XX[18]/47,XX,i(9)(p10),+mar[1]	ND	JAK2	AP/BP
6	67	ET	F	BM	48,XX,+8,+8[20]	8p23.3q24.3(0_146293435)x4, 9p24.3p21.3(0_24234310)x2 hmz	JAK2, IDH1, SRSF2	AP/BP
7	51	ET	M	BM	46,XY,del(15)(q15q23),t(17;18)(p12;q11.2) [7]/46,XY[13]	ND	CALR, TET2	MF
8	45	ET	F	PB	46,XX,del(13)(q12q14)[4]/ 46,XX,del(13)(q12q32)[4]/ 46,XX,dup(1)(q21q42)[3]/ 48,XX,+der(1)t(1;19)(p12;p12),+8[2]/ 46,XX[7]	ND	Triple Negative, SH2B3	MF
9	39	ET	F	PB	46,XX,del(13)(q12q14)[8]/ 46,XX[7]	ND	Triple Negative	MF
10	80	ET	M	BM	45,X,-Y[16]/46,idem,+8[3]/ 45,XY,-3[1]	ND	JAK2, SRSF2	No Progression
11	74	ET	F	BM	46,XX,del(20)(q11.2q13.3)[11]/ 46,XX[9]	ND	JAK2, TET2	MF
12	54	ET	M	PB	47,XY,+Y[20]	Yp11.32q12(0_59373566)x3	JAK2	No Progression
13	81	ET	M	#N/A	45,X,-Y[18]/ 46,XY[2]	Yp11.32q12(0_59373566)x0	JAK2	No Progression
14	73	ET	M	BM	45,X,-Y[7]/ 46,XY[22]/ 46,XY,?del(8)(q22)[1]	ND	Not Performed	No Progression
15	38	ET	M	PB	46,XY,+9,-14[1]/ 46,XY[6]	9p24.3q34.3(0_141213431)x3	JAK2	MF
16	70	ET	M	BM	46,XY,der(6)t(3;6)(q25;p22)[20]	Xq26.2q26.3(133506194_134184567)x0, 1p36.13p22.1(1089699_93720070)x2 hmz, 3q25.32q29(157780129_197861598)x3, 6p25.3p22.1(204009_27534514)x1	MPL, TET2	MF
17	83	ET	F	BM	46,XX,del(13)(q12q14)[3]/ 46,XX[17]	ND	JAK2	No Progression
18	72	ET	F	BM	46,XX,del(20)(q11.2q13.3)[1]/ 46,XX[19]	9p24.3p13.1(0_38694064)x2 hmz	JAK2, DNMT3A	MF
19	89	ET	M	BM	46,XY,del(13)(q12q14)[5]/ 46,XY,del(13)(q12q22)[3]/ 46,XY[12]	ND	MPL	MF
20	67	ET	F	PB	46,XX,der(6)t(1;6)(q21;p25),t(9;11)(p24;q21),del(13)(q12q22)[1]/ 46,idem,del(11)(q14q23)[8]/ 46,idem,del(12)(p13p11.2)[7]/ 46,XX,t(11;17)(p15;q11.2)[4]	ND	JAK2, DNMT3A, EZH2	AP/BP
21	81	ET	M	BM	46,XY,t(9;22)(q34;q11.2)[20]	ND	JAK2	CML
22	52	ET	M	BM	46,XY,del(13)(q12q22)[9]/ 46,XY[11]	ND	JAK2, GATA1	MF
90	65	prePMF	F	BM	45,XX,del(12)(p11.2p13),-18[1]/ 46,XX[17]	ND	JAK2, RUNX1, SF3B1*, STAG2	MDS
156	64	ET	M	BM	47,XY,+9[4]/ 46,XY[15]/ 47,XY,+18[1]	ND	JAK2	PV
163	77	ET	F	BM	46,XX,dup(1)(q21q32)[8]/ 46,XX[12]	1q21.2q32.1(150189472_204513328)x3, 5q21.3q23.1(107501113_115990907)x1	CALR	No Progression
167	80	ET	M	BM	46,XX,del(20)(q11.2q13.1)[4]/ 46,XX[16]	9p24.3p22.1(0_19585456)x2 hmz, 17p11.2(17803134_19293234)x1~2, 20q11.23q13.2(34975018_50468054)x1	JAK2	No Progression
169	77	ET	M	BM	46,XY,del(20)(q11.2q13.3)[14]/ 46,XY[6]	9p24.3p24.1(0_6651634)x2 hmz, 20q11.21q13.32(32060886_58171738)x1, Yp11.32q12(0_59373566)x0	JAK2	MF

## Data Availability

The datasets used and/or analyzed in the study are available from the corresponding authors upon request.
